# DNA Damage-Inducible Pyocin Expression Is Independent of RecA in *xerC*-Deleted Pseudomonas aeruginosa

**DOI:** 10.1128/spectrum.01167-22

**Published:** 2022-06-16

**Authors:** Adam S. Bronson, Nina S. Baggett, Matthew T. Cabeen

**Affiliations:** a Department of Microbiology and Molecular Genetics, Oklahoma State University, Stillwater, Oklahoma, USA; Penn State University

**Keywords:** *Pseudomonas aeruginosa*, RecA, XerC, antimicrobial peptides, pyocins

## Abstract

Pyocins are interbacterial killing complexes made by Pseudomonas aeruginosa primarily to enact intraspecific competition. DNA damage and the ensuing activation of RecA initiate canonical pyocin expression. We recently discovered that deletion of *xerC*, which encodes a tyrosine recombinase involved in chromosome decatenation, markedly elevates basal pyocin production independently of RecA. Interestingly, the already-elevated basal pyocin expression in Δ*xerC* cells is substantially further increased by ciprofloxacin treatment. Here, we asked whether this further increase is due to DNA damage additionally activating the canonical RecA-dependent pyocin expression pathway. We also interrogated the relationship between XerC recombinase activity and pyocin expression. Surprisingly, we find that DNA damage-induced pyocin stimulation in Δ*xerC* cells is independent of RecA but dependent on PrtN, implying a RecA-independent means of DNA damage sensing that activates pyocin expression via PrtN. In sharp contrast to the RecA independence of pyocin expression in Δ*xerC* strains, specific mutational inactivation of XerC recombinase activity (XerC_Y272F_) caused modestly elevated basal pyocin expression and was further stimulated by DNA-damaging drugs, but both effects were fully RecA dependent. To test whether pyocins could be induced by chemically inactivating XerC, we deployed a previously characterized bacterial tyrosine recombinase inhibitor. However, the inhibitor did not activate pyocin expression even at growth-inhibitory concentrations, suggesting that its principal inhibitory activity resembles neither XerC absence nor enzymatic inactivation. Collectively, our results imply a second function of XerC, separate from its recombinase activity, whose absence permits RecA-independent but DNA damage-inducible pyocin expression.

**IMPORTANCE** The opportunistic pathogen Pseudomonas aeruginosa produces pyocins—intraspecific, interbacterial killing complexes. The canonical pathway for pyocin production involves DNA damage and RecA activation. Pyocins are released by cell lysis, making production costly. We previously showed that cells lacking the tyrosine recombinase XerC produce pyocins independently of RecA. Here, we show that DNA-damaging agents stimulate pyocin expression in Δ*xerC* strains without involving RecA. However, strains mutated for XerC recombinase activity display strictly RecA-dependent pyocin production, and a known bacterial tyrosine recombinase inhibitor does not elicit pyocin expression. Our results collectively suggest that the use of XerC inhibition as an antipseudomonal strategy will require targeting the second function of XerC in regulating noncanonical pyocin production rather than targeting its recombinase activity.

## INTRODUCTION

The impressive interbacterial competitive arsenal of Pseudomonas aeruginosa includes the R-type pyocins, phage tail-like protein complexes that target other P. aeruginosa strains and kill them via a contractile mechanism (1–5). Like the phages they resemble, pyocins escape producer cells via lysis, thanks to holin and lysin enzymes encoded in the pyocin gene cluster (6). Hence, making pyocins imposes a cost on the producer population. The canonical pathway for pyocin expression is initiated by DNA damage, which activates RecA and also leads to the SOS response (7, 8). Active RecA stimulates autocleavage of a repressor called PrtR, relieving repression of *prtN*, which encodes an activator of pyocin gene cluster expression (9). Because of this RecA-mediated genetic logic, agents like the fluoroquinolone antibiotic ciprofloxacin and mitomycin C (MMC; a DNA cross-linker) activate pyocin expression (2, 10). The subsequent lysis of pyocin-producing cells appears to be one way that fluoroquinolones kill P. aeruginosa cells, as cells mutant for *recA* or pyocin genes show greater resistance to such antibiotics (11). We recently discovered that strains lacking the tyrosine recombinase XerC exhibit markedly elevated basal pyocin expression that is independent of RecA (12). The overproduced pyocins released by Δ*xerC* strains are effective in killing sensitive strains, and *xerC* complementation restores wild-type levels of pyocin production (12). Interestingly, we also found that treatment of Δ*xerC* cells with ciprofloxacin resulted in a substantial further increase in pyocin expression over their already-elevated basal levels (12). One attractive explanation for this further stimulation might be that ciprofloxacin treatment additionally activates the DNA damage-inducible canonical pathway for pyocin expression, so that RecA-dependent and RecA-independent pathways for pyocin expression are simultaneously active.

In both P. aeruginosa and Escherichia coli, XerC acts with a second recombinase, XerD, to catalyze site-specific recombination at chromosomal *dif* sites to decatenate replicated chromosomes (13–16). We found that genetic inactivation of XerC recombinase activity via Phe substitution for the nucleophilic Tyr residue required for DNA cleavage and subsequent recombination (17) at the active site (XerC_Y272F_) increased pyocin expression, but to a substantially lesser degree than the full *xerC* deletion (12). This finding suggested that loss of recombinase activity contributes to but does not fully explain the elevated pyocin expression of strains deleted for *xerC*. The role of XerC recombinase activity in the context of elevated pyocin expression is not yet known. Does blocking recombinase activity activate the canonical or noncanonical pathways of pyocin expression? Further, elevated pyocin expression comes at a fitness cost, as Δ*xerC* strains grow more poorly than their wild-type counterparts and are more sensitive to antibiotics like ciprofloxacin (12). Hence, if XerC recombinase activity could be chemically inhibited to increase pyocin expression and sensitize cells to fluoroquinolone antibiotics, a recombinase inhibitor might have clinical utility when used in combination with fluoroquinolones.

Here, we address four questions about the pathways by which DNA-damaging drugs stimulate pyocin expression and their relationship to XerC recombinase activity. We first ask whether DNA damage-mediated induction of pyocin expression in Δ*xerC* cells is mediated by the canonical RecA-dependent pathway and whether the pyocin expression activator PrtN is required. Next, we examine whether the very high level of pyocin expression in drug-treated cells remains heterogeneous at the single-cell level. We then ask whether pyocin induction in strains inactivated for XerC recombinase activity occurs independently of RecA, as in Δ*xerC* strains. Finally, we ask how a previously characterized antibacterial Holliday junction-binding, tyrosine recombinase-inhibiting hexapeptide (18–20) impacts P. aeruginosa cell growth and/or pyocin expression. Our findings reveal that, surprisingly, DNA damage-induced pyocin expression in Δ*xerC* cells is independent of RecA but remains dependent on PrtN. Even strongly expressing cell populations maintain their heterogeneity across individual cells. In sharp contrast to *xerC* deletion, XerC enzymatic inactivation appears to exclusively stimulate the canonical, RecA-dependent pyocin activation pathway, suggesting a second, recombination-independent function for XerC in pyocin regulation. Finally, the hexapeptide tyrosine recombinase inhibitor can impede growth of P. aeruginosa but does not stimulate pyocin expression, suggesting that its primary mode of growth inhibition does not resemble XerC genetic inactivation or deletion.

## RESULTS

### RecA-independent stimulation of pyocin expression by ciprofloxacin in a *xerC* deletion background.

**FIG 1:**
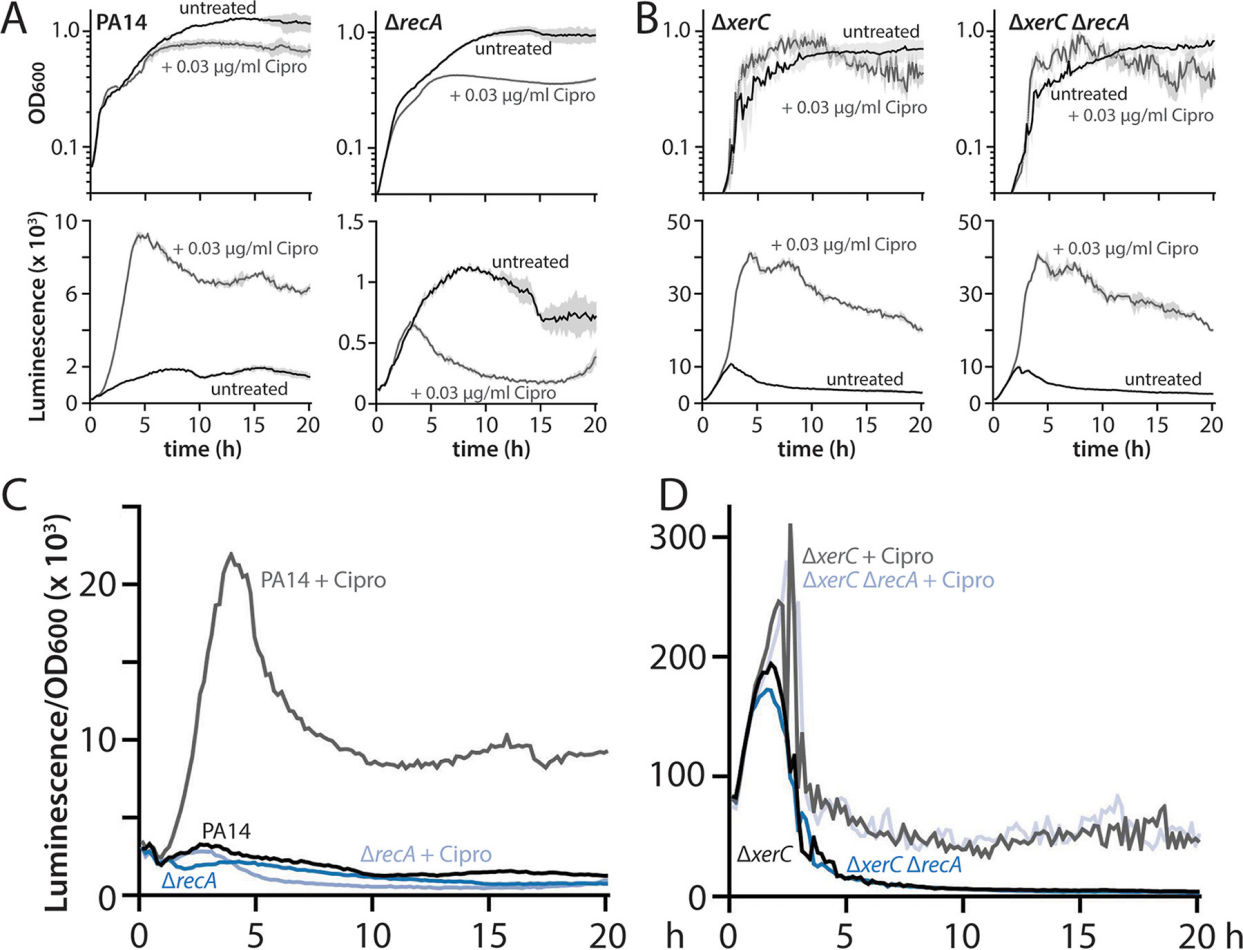
Ciprofloxacin-mediated stimulation of pyocin expression in Δ*xerC* strains is RecA independent. (A) Representative growth curves (OD_600_) and luminescence traces (P*_07990_*-*lux*) of wild-type PA14 (MTC2280) and Δ*recA* (MTC2302) strains treated (gray) or not (black) with 0.03 μg/mL ciprofloxacin (Cipro). Note that the *y* axis scales on the luminescence graphs vary. Light gray shading surrounding the traces indicates standard deviation from three technical replicates. Time is indicated in hours. (B) Representative growth curves and luminescence traces as in panel A, but for Δ*xerC* (MTC2297) and Δ*xerC* Δ*recA* (MTC2301) strains. (C and D) OD-normalized luminescence traces of the indicated strains from panels A and B, respectively.

Ciprofloxacin is a known inducer of pyocin expression via the canonical RecA-dependent pathway for pyocin expression. In this pathway, ciprofloxacin treatment blocks gyrase activity and causes DNA damage, thereby activating RecA; active RecA stimulates cleavage of PrtR, a repressor of *prtN* (9). The resulting derepression of *prtN*, which encodes an activator of pyocin gene expression, causes elevated pyocin production. We first confirmed the RecA dependence of ciprofloxacin-induced pyocin expression in wild-type cells bearing a luminescent reporter for R/F pyocin expression. Treatment with a sublethal concentration of ciprofloxacin (0.03 μg/mL) strongly stimulated pyocin expression, and deletion of *recA* abolished ciprofloxacin-stimulated pyocin expression (Fig. 1A and C; note the different *y* axis scales for luminescence graphs), consistent with the canonical model. We previously reported that a Δ*xerC* strain not only displayed markedly elevated pyocin expression relative to the wild type but also showed a substantial further increase in pyocin expression upon ciprofloxacin treatment (12). As the elevated basal expression of pyocins in Δ*xerC* strains is independent of the canonical RecA-mediated pathway, we reasoned that the further increase in pyocin expression stimulated by ciprofloxacin in Δ*xerC* cells might be due to simultaneous activation of the canonical, RecA-dependent pathway. To test this notion, we treated Δ*xerC* Δ*recA* cells with ciprofloxacin and examined pyocin expression. Ciprofloxacin-stimulated pyocin expression in Δ*xerC* cells occurred irrespective of the presence of RecA, with indistinguishable phenotypes (Fig. 1B and D). This result indicated that the further elevation of pyocin expression under ciprofloxacin treatment in a Δ*xerC* background is not due to additional activation of the canonical, RecA-dependent pathway. Instead, the ciprofloxacin-mediated increase of pyocin expression in Δ*xerC* cells occurs independently of RecA.

### RecA-independent stimulation of pyocin expression in Δ*xerC* cells by mitomycin C.

**FIG 2:**
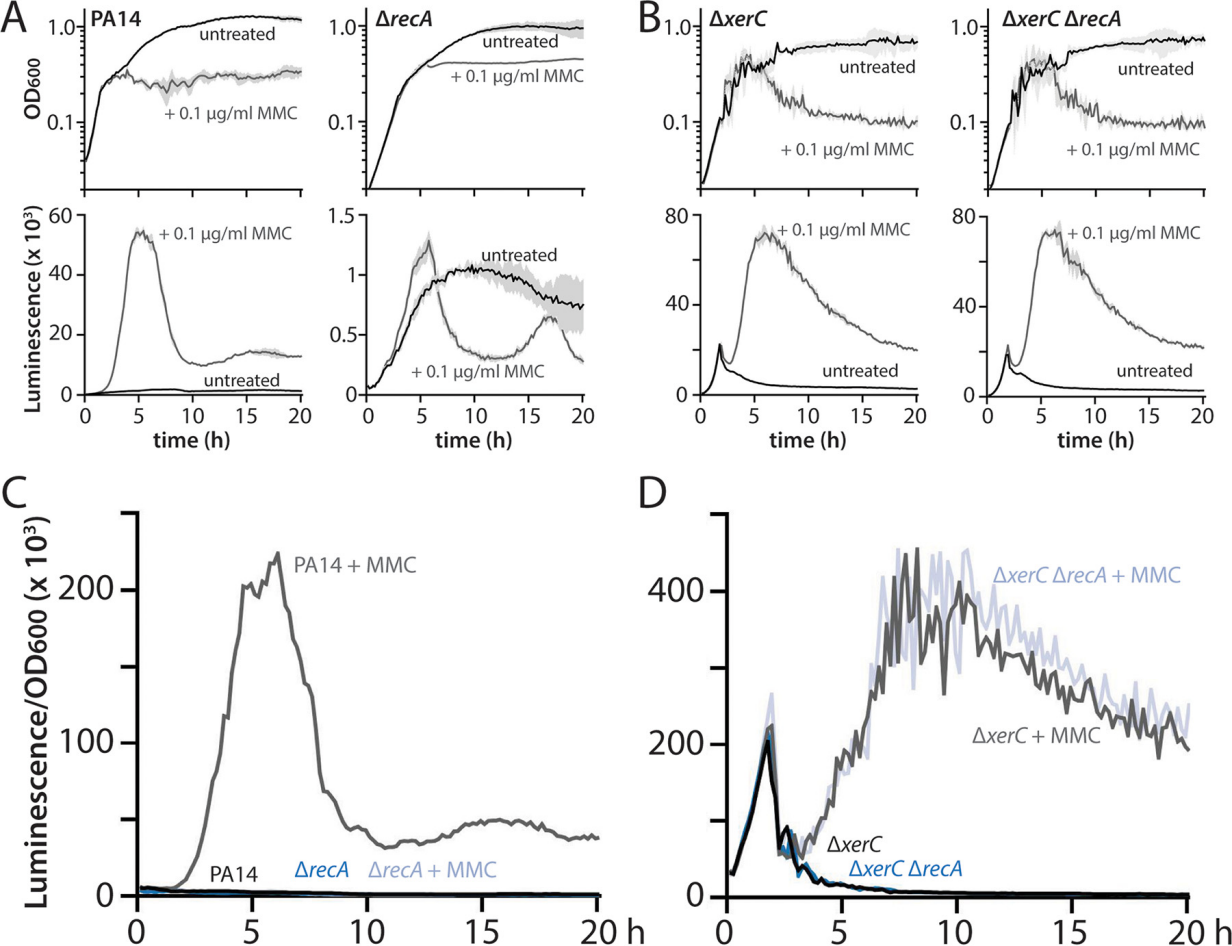
Mitomycin C-mediated stimulation of pyocin expression in Δ*xerC* strains is RecA independent. (A) Representative growth curves (OD_600_) and luminescence traces (P*_07990_-lux*) of wild-type PA14 (MTC2280) and Δ*recA* (MTC2302) strains treated (gray) or not (black) with 0.1 μg/mL mitomycin C (MMC). Note that the *y* axis scales on the luminescence graphs vary. Light gray shading surrounding the traces indicates standard deviation from three technical replicates. Time is indicated in hours. (B) Representative growth curves and luminescence traces as in panel A, but for Δ*xerC* (MTC2297) and Δ*xerC* Δ*recA* (MTC2301) strains. (C and D) OD-normalized luminescence traces of the indicated strains from panels A and B, respectively.

We next asked whether treatment with mitomycin C (MMC) would show the same pattern of RecA dependence as ciprofloxacin. MMC differs from ciprofloxacin as it directly damages DNA (it is a DNA cross-linking agent), and it is a known strong activator of pyocin expression in P. aeruginosa (2, 10). When we treated wild-type cells with 0.1 μg/mL MMC, which inhibited cell growth to a slightly greater degree than 0.03 μg/mL ciprofloxacin (Fig. 1A and Fig. 2A), we observed strong pyocin expression that peaked at approximately 10-fold that induced by ciprofloxacin on an optical density (OD)-normalized basis (Fig. 1A and C and Fig. 2A and C; note the different *y* axis scales for luminescence graphs). As with ciprofloxacin, deletion of *recA* fully abolished this very strong MMC-stimulated pyocin induction. MMC treatment also strongly increased pyocin expression in Δ*xerC* cells, with a later, but approximately 2-fold stronger, peak of expression than in untreated cells (Fig. 2B and D). This MMC-mediated increase, as with ciprofloxacin, was independent of RecA (Fig. 2B and D). Collectively, our results indicate that in Δ*xerC* cells, pyocin expression occurs via a RecA-independent pathway that, surprisingly, is strongly inducible by DNA-damaging agents.

### The RecA-dependent and RecA-independent pathways for pyocin expression both require PrtN.

**FIG 3:**
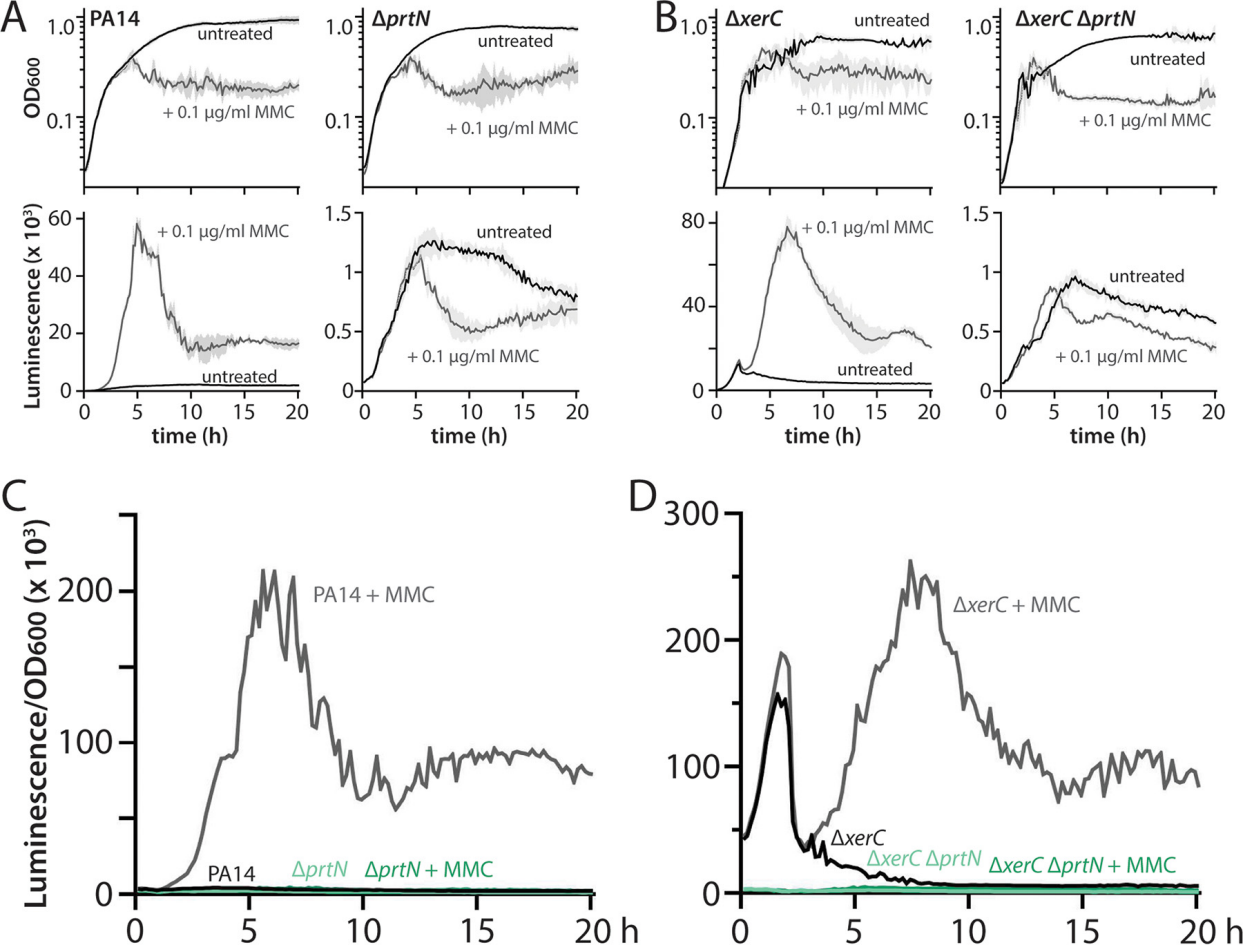
Mitomycin C-mediated stimulation of pyocin expression in wild-type and Δ*xerC* strains requires PrtN. (A) Representative growth curves (OD_600_) and luminescence traces (P*_07990_-lux*) of wild-type PA14 (MTC2280) and Δ*prtN* (MTC2303) strains treated (gray) or not (black) with 0.1 μg/mL mitomycin C (MMC). Note that the *y* axis scales on the luminescence graphs vary. Light gray shading surrounding the traces indicates standard deviation from three technical replicates. Time is indicated in hours. (B) Representative growth curves and luminescence traces as in panel A, but for Δ*xerC* (MTC2297) and Δ*xerC* Δ*prtN* (MTC2298) strains. (C and D) OD-normalized luminescence traces of the indicated strains from panels A and B, respectively.

We previously showed that the elevated basal expression of pyocins in Δ*xerC* strains required the pyocin expression activator PrtN (12), suggesting that both the canonical (RecA-dependent) and noncanonical (RecA-independent) pathways for pyocin expression share a requirement for PrtN to enact pyocin expression. We thus asked whether PrtN was also required for the further stimulation of pyocin expression in cells undergoing DNA damage. When we challenged wild-type and Δ*xerC* cells deleted or not for *prtN* with 0.1 μg/mL MMC, we saw that, as expected, deletion of *prtN* abolished the ability of MMC to stimulate pyocin expression in a wild-type background (Fig. 3A and C; note the different *y* axis scales for luminescence graphs). Furthermore, *prtN* deletion also fully abrogated pyocin expression in a Δ*xerC* background, even under MMC treatment (Fig. 3B and D). These results confirm the common requirement for PrtN for P. aeruginosa cells to activate pyocin expression, irrespective of which pathway is active.

### RecA-independent pyocin stimulation is heterogeneous at the single-cell level.

**FIG 4:**
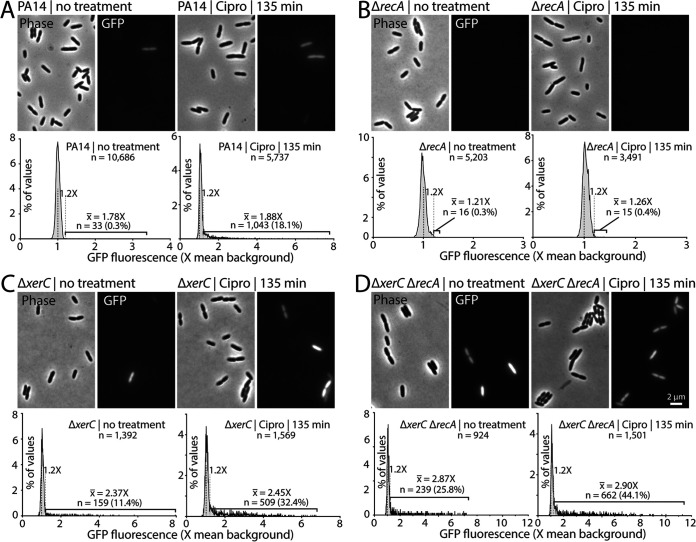
Single-cell analysis of pyocin expression in ciprofloxacin-induced strains. Representative phase-contrast and GFP fluorescence (P*_07990_-gfp*) micrographs are shown in each panel above distributions of mean GFP fluorescence in individual cells of the indicated strains. As in our previous work, cells above a cutoff of 1.2× (gray dashed line) background fluorescence (black dashed line) were considered GFP positive. In each panel, untreated cells are compared to the same strain treated with 0.03 μg/mL ciprofloxacin for 135 min. All micrographs are sized and scaled identically. (A) PA14 (MTC2277). (B) Δ*recA* strain (MTC2448). (C) Δ*xerC* strain (MTC2252). (D) Δ*xerC* Δ*recA* strain (MTC2291). Percentages and average fluorescence (× background) of GFP-positive cells are indicated. A larger number of cells was analyzed in strains expected to have a lower proportion of GFP positivity to improve detection of rare GFP-positive cells.

Pyocin expression, whether induced in wild-type cells via the canonical RecA-dependent pathway by ciprofloxacin or induced noncanonically in Δ*xerC* cells, shows strong heterogeneity at the single-cell level (12). Most cells showed undetectable pyocin expression (pyocin-OFF), whereas a subset of cells (pyocin-ON) displayed strong pyocin expression (visualized as a green fluorescent protein [GFP] transcriptional reporter driven by the *PA14_07990* promoter at the beginning of the R/F pyocin gene cluster). We further showed that pyocin-ON cells most often showed progressively increasing GFP fluorescence until cells explosively lysed due to the holin- and lysin-encoding genes in the R/F pyocin cluster (12). In the present work, bulk assays showed extremely strong levels of pyocin expression when Δ*xerC* cells were treated with MMC or ciprofloxacin (Fig. 1 and 2). Such an increase in overall expression might be due either to a general increase in gene expression across all cells or to an increase in the portion of pyocin-ON cells within a population of mainly pyocin-OFF cells. We thus asked whether heterogeneity in pyocin expression was preserved in wild-type or Δ*xerC* cells deleted or not for *recA* and treated or not with 0.03% ciprofloxacin for 135 min. Notably, this treatment concentration and duration minimally impacted cell growth in our bulk assays (Fig. 1A and B). Our control strains were concordant with our previous results (12): untreated wild-type PA14 cells showed very few (0.3%) GFP-positive cells that were relatively dim, whereas 135-min treatment with 0.03 μg/mL ciprofloxacin substantially increased the proportion of GFP-positive cells to 18.1% (Fig. 4A). Notably, even under ciprofloxacin treatment, the great majority of cells showed no detectable GFP fluorescence. Consistent with our bulk assay data, a Δ*recA* strain also showed very few pyocin-ON cells, irrespective of ciprofloxacin treatment (Fig. 4B). The few cells that exceeded our threshold for GFP positivity were only just above the threshold (Fig. 4B), consistent with an overall lack of pyocin expression and hence a strict dependence on RecA for pyocin expression in a *xerC*^+^ genetic background.

In both Δ*xerC* and Δ*xerC* Δ*recA* strain backgrounds, untreated cells showed the expected strong heterogeneity and substantially increased numbers of pyocin-ON cells (Fig. 4C and D). Treatment of either strain with ciprofloxacin markedly increased the proportion of pyocin-ON cells to at least one-third of the total cells observed without substantially changing their average GFP brightness; both strains still exhibited strong heterogeneity, with most cells showing no detectable GFP fluorescence (Fig. 4C and D). For both treated and untreated cells, we observed more fluorescent cells and slightly greater average fluorescence in the Δ*xerC* Δ*recA* double mutant (Fig. 4C and D), clearly indicating that loss of RecA does not impair pyocin expression in Δ*xerC* cells.

### Inactivation of XerC recombinase activity induces pyocin expression solely via RecA-dependent mechanisms.

**FIG 5:**
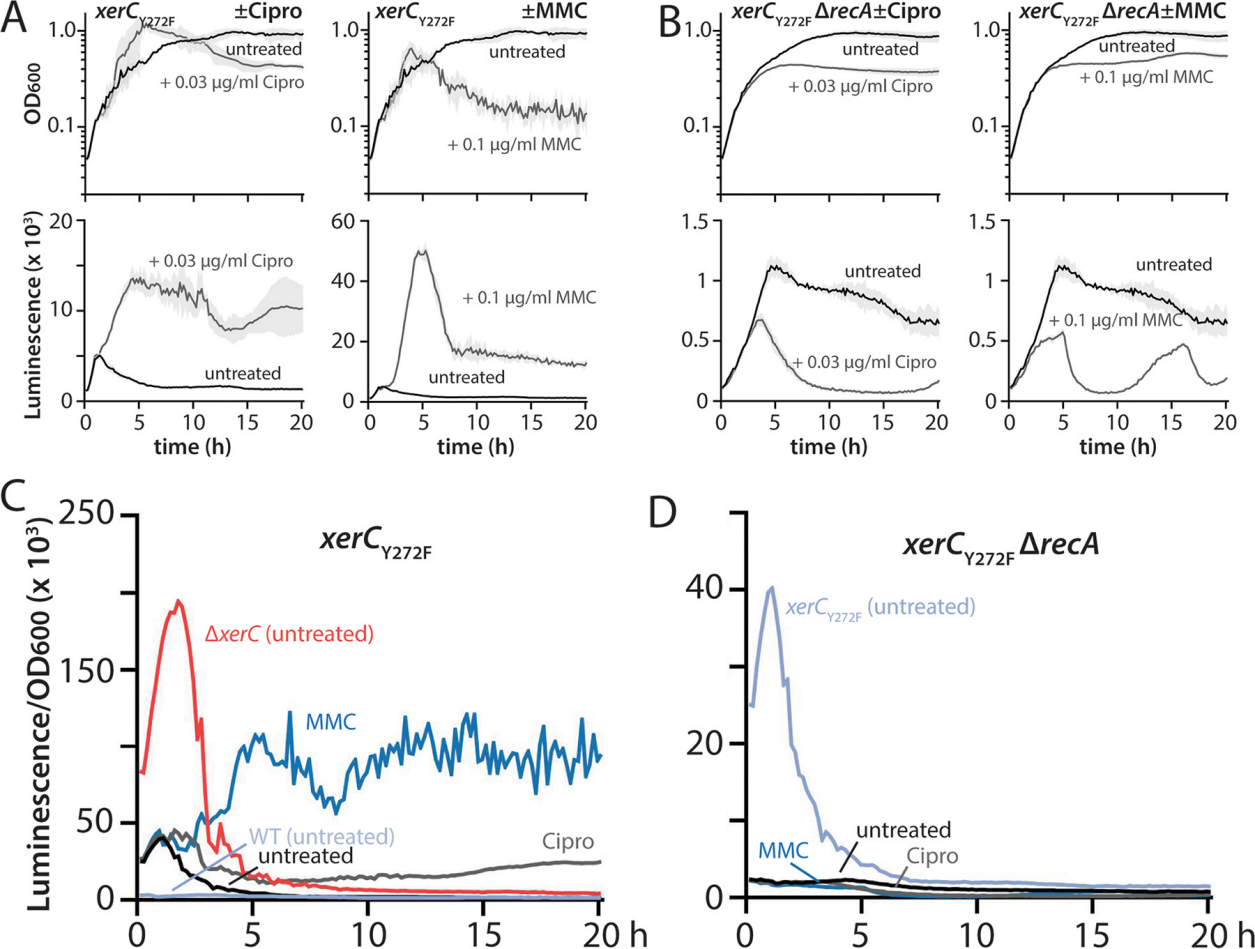
Pyocin expression in cells with catalytically inactive XerC is RecA dependent. (A) Representative growth curves (OD_600_) and luminescence traces (P*_07990_-lux*) of *xerC*_Y272F_ (MTC2339) treated (gray) or not (black) with 0.03 μg/mL ciprofloxacin (Cipro) or 0.1 μg/mL mitomycin C (MMC). Note that the *y* axis scales on the luminescence graphs vary. Light gray shading surrounding the traces indicates standard deviation from three technical replicates. Time is indicated in hours. (B) Representative growth curves and luminescence traces as in panel A, but for *xerC*_Y272F_ Δ*recA* (MTC2444). (C and D) OD-normalized luminescence traces of the indicated strains from panels A and B, respectively, together with other strains shown for reference.

Because we observed that both basal and DNA damage-induced pyocin expression in Δ*xerC* strains occurred independently of RecA, we next asked whether the same were true of *xerC*_Y272F_ strains bearing only a recombinase-inactive version of XerC. Consistent with our previous results (12), a *xerC*_Y272F_ strain showed an intermediate phenotype, with much greater pyocin expression than the wild type but roughly 5-fold less than in a Δ*xerC* strain (Fig. 5A and C; note the different *y* axis scales for luminescence graphs). While pyocin expression in *xerC*_Y272F_ strains was stimulated by both ciprofloxacin and MMC, the degree of stimulation was modest, with only slightly more pyocin expression in ciprofloxacin-treated *xerC*_Y272F_ cultures than in ciprofloxacin-treated wild-type cultures (Fig. 5C, compare with Fig. 1C). Moreover, MMC treatment resulted in less expression in *xerC*_Y272F_ cultures than in the wild type (Fig. 5C, compare with Fig. 2C), a distinct departure from the dramatic increases in pyocin expression upon ciprofloxacin or MMC treatment of Δ*xerC* strains (Fig. 1D and Fig. 2D).

We then examined the role of RecA in the elevated basal pyocin expression of *xerC*_Y272F_ strains and its further stimulation by ciprofloxacin or MMC. Strikingly, deletion of *recA* not only abolished ciprofloxacin- and MMC-mediated stimulation of pyocin expression, it also fully abolished the elevated basal pyocin expression of the *xerC*_Y272F_ strain (Fig. 5B and D; note the different *y* axis scales from Fig. 5A for luminescence graphs), reducing it to levels indistinguishable from those of a Δ*recA* strain (Fig. 1A). We interpret these data as indicating that enzymatic inactivation of XerC recombinase activity provokes pyocin expression exclusively via the canonical RecA-dependent pathway. The more modest effect of ciprofloxacin and MMC on pyocin expression in a *xerC*_Y272F_ background is consistent with this interpretation, as the RecA-mediated DNA damage-response pathways typically stimulated by these agents would already be partially active. Furthermore, we reproducibly observed that MMC- and especially ciprofloxacin-treated *xerC*_Y272F_ cultures initially outgrew both untreated control cultures (Fig. 5A, top row) and drug-treated wild-type cells (Fig. 1A and Fig. 2A). This phenomenon is in accord with “preactivation” of RecA in *xerC*_Y272F_ cells providing a measure of protection against DNA damage-inducing drugs. Conversely, the greater OD of the *xerC*_Y272F_ Δ*recA* culture than the *xerC*_Y272F_ parent under MMC treatment at later time points (compare Fig. 5A and B, top right panels) likely reflects reduced cell lysis because cells are no longer producing pyocins. Importantly, these results show that loss of XerC recombinase activity and absence of XerC induce pyocin expression via separate mechanisms. The RecA-independent pyocin stimulation in Δ*xerC* strains cannot be attributed to loss of XerC recombinase activity, thereby implying a second, RecA-independent function of XerC in the regulation of pyocin expression.

### A recombinase inhibitor peptide inhibits growth but does not stimulate pyocin expression.

**FIG 6:**
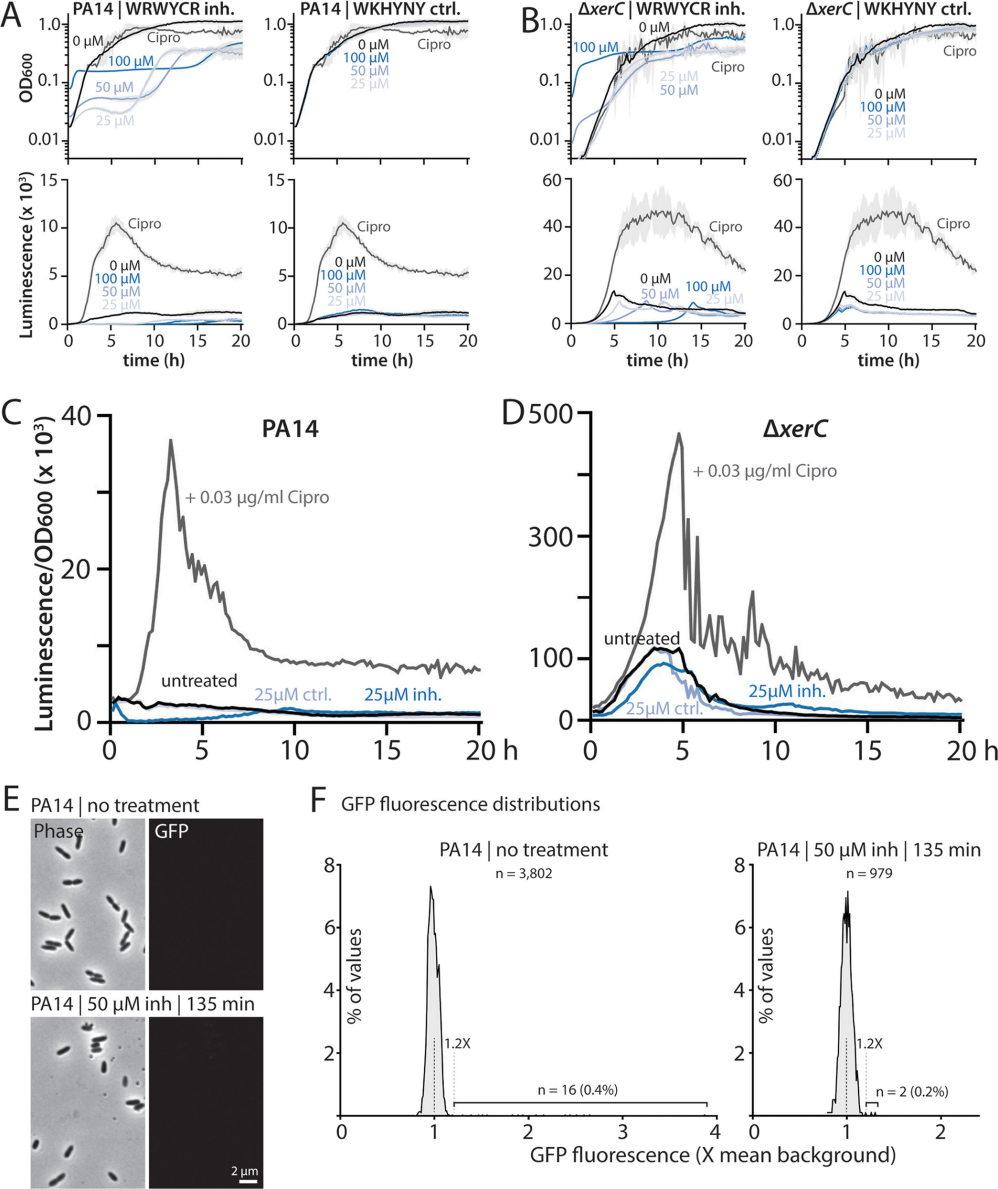
Hexapeptide recombinase inhibitors do not stimulate pyocin expression. (A) Representative growth curves (OD_600_) and luminescence traces (P*_07990_-lux*) of wild-type PA14 (MTC2280) treated with the indicated concentrations of WRWYCR recombinase inhibitor peptide or with WKHYNY, a control peptide with no inhibitor activity (blue shades). Cells were also treated with 0.03 μg/mL ciprofloxacin (Cipro) as a control (gray). Light gray shading surrounding the traces indicates standard deviation from three technical replicates. Time is indicated in hours. (B) Representative growth curves and luminescence traces as in panel A, but for Δ*xerC* (MTC2297). (C and D) OD-normalized luminescence traces of the indicated strains and treatments from panels A and B, respectively. (E) Representative phase-contrast and GFP fluorescence (P_07990_*-gfp*) micrographs of wild-type PA14 (MTC2277) cells treated or not with 50 μM WRWYCR inhibitor peptide for 135 min. (F) Distributions of mean GFP fluorescence in individual cells treated as in panel E. As in our previous work, cells above a cutoff of 1.2× (gray dashed line) background fluorescence (black dashed line) were considered GFP positive.

Given that both specific recombinase inhibition of XerC and full *xerC* deletion increase pyocin expression, albeit via different mechanisms, we inquired whether a known tyrosine recombinase inhibitor could elicit pyocin expression. Drug treatment that inhibited XerC to stimulate pyocin expression would likely sensitize cells to fluoroquinolones like ciprofloxacin, imbuing recombinase inhibitors with potential therapeutic utility. Known inhibitors include hexapeptides that bind to Holliday junctions to achieve tyrosine recombinase inhibition (18); these inhibitors also inhibit growth of E. coli cells (19). However, these hexapeptide inhibitors have not been tested for their ability to stimulate pyocin production in P. aeruginosa. We treated wild-type cells with the inhibitor WRWYCR (19) at concentrations ranging from 25 to 100 μM, all of which markedly impaired P. aeruginosa growth (Fig. 6A). In contrast, the control hexapeptide WKHYNY (19) showed no inhibition of growth at the same treatment concentrations (Fig. 6A). Neither the WRWYCR inhibitor nor the WKHYNY control elicited pyocin expression beyond the level of untreated cells (Fig. 6A and C), indicating that the inhibitor does not affect XerC in a way that provokes pyocin expression. Next, we applied the same treatments to Δ*xerC* cells, reasoning that this genetic background might sensitize cells to pharmacological inhibition of other recombinases. The growth of Δ*xerC* cells was inhibited by WRWYCR but not WKHYNY, with only minor differences from the wild type (Fig. 6B). Moreover, no differences in pyocin expression were observed under either inhibitor or control peptide treatment (Fig. 6B and D).

As a second test of the effect of inhibitor peptides, we also examined microscopically wild-type pyocin-GFP reporter cells treated with a 50 μM concentration of the WRWYCR inhibitor, a concentration that substantially inhibited growth (Fig. 6A). The peptide-treated cells appeared morphologically similar to untreated cells, although we observed a qualitative decrease in the number of dividing cells (Fig. 6E), consistent with the growth inhibition we measured in bulk (Fig. 6A). In accord with our bulk measurements, peptide treatment did not elicit GFP fluorescence (Fig. 6F), further supporting the conclusion that the peptide recombinase inhibitor WRWYCR does not stimulate pyocin production in P. aeruginosa.

### Subinhibitory concentrations of peptide inhibitors do not stimulate pyocin expression.

**FIG 7:**
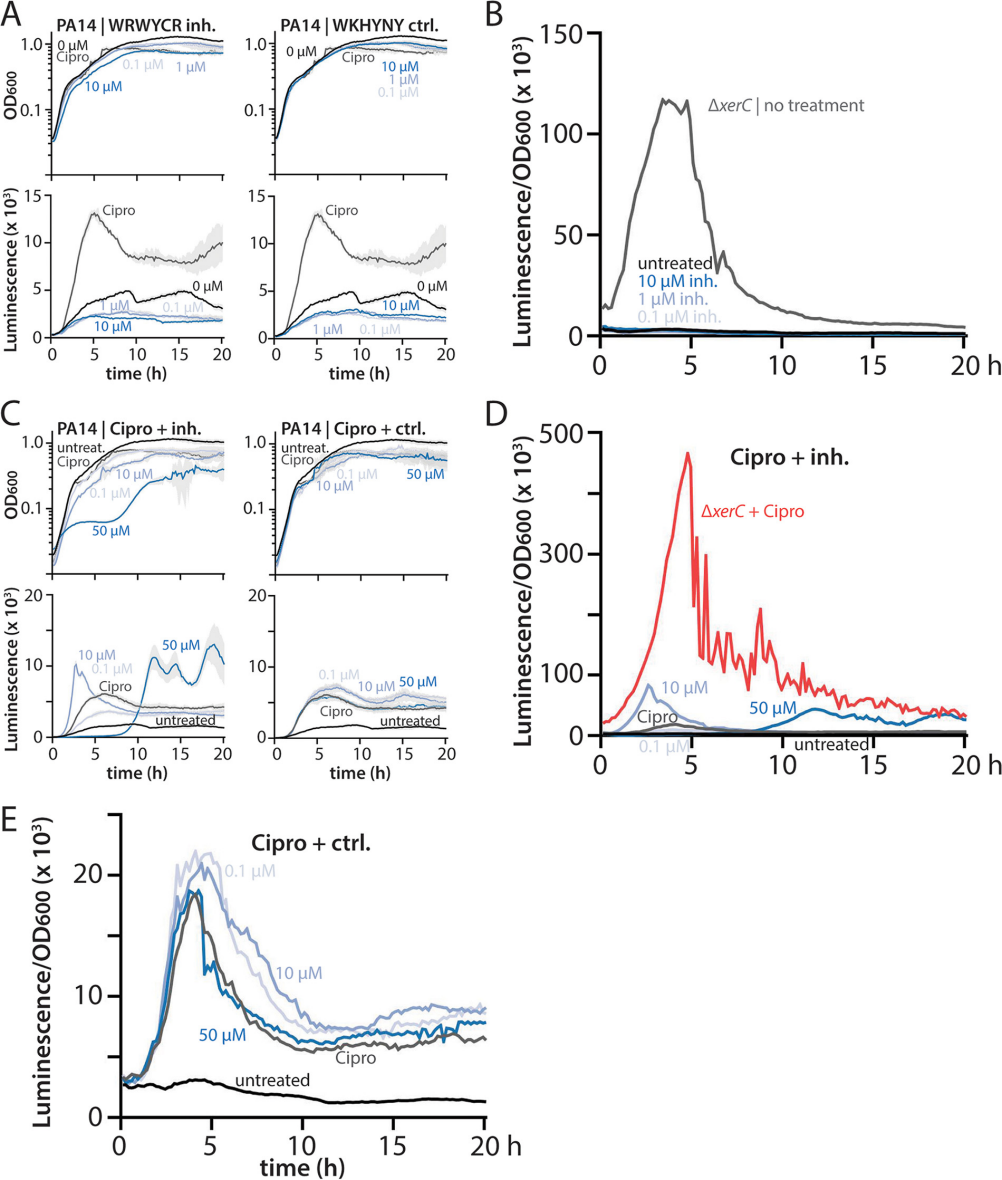
Recombinase inhibitors do not stimulate pyocin expression at subinhibitory concentrations or in combination with ciprofloxacin. (A) Representative growth curves (OD_600_) and luminescence traces (P*_07990_-lux*) of wild-type PA14 (MTC2280) treated with the indicated concentrations of WRWYCR recombinase inhibitor peptide or with WKHYNY, a control peptide with no inhibitor activity (blue shades). Cells were also treated with 0.03 μg/mL ciprofloxacin (Cipro) as a control (gray). Light gray shading surrounding the traces indicates standard deviation from three technical replicates. Time is indicated in hours. (B) OD-normalized luminescence traces of the indicated treatments from panel A. (C) Representative growth curves (OD_600_) and luminescence traces (P_07990_*-lux*) of wild-type PA14 (MTC2280) treated with 0.03 μg/mL ciprofloxacin alone (gray) or with the indicated concentrations of WRWYCR inhibitor or WKHYNY control peptides (blue shades). Light gray shading surrounding the traces indicates standard deviation from three technical replicates. Time is indicated in hours. (D and E) OD-normalized luminescence traces of the indicated treatments from panel C. In panel D, ciprofloxacin-treated Δ*xerC* cells (MTC2297) are shown as a reference for the degree of pyocin induction in the absence of XerC.

It was clear from our data that growth-inhibitory concentrations of the tyrosine recombinase inhibitor WRWYCR did not stimulate pyocin expression (Fig. 6). However, our work with ciprofloxacin and MMC, which strongly elicit pyocin expression even at concentrations that do not fully inhibit cell growth, prompted us to examine the effects of lower concentrations of hexapeptides that have only minor effects on growth. In these experiments, we treated wild-type cells with 0.1 to 10 μM WRWYCR or the WKHYNY control peptide. Treatment with 10 μM inhibitor affected culture growth to a similar degree as did 0.03 μg/mL ciprofloxacin, whereas lower concentrations of inhibitor had correspondingly smaller effects, as did the control peptide (Fig. 7A). Treatment with low concentrations of WRWYCR resulted in no increase in pyocin expression over that in untreated cells (Fig. 7A and B).

### Coadministration of peptide inhibitors to ciprofloxacin-treated cells modestly impacts pyocin expression.

As a final test of the impact of recombinase inhibitor peptides on pyocin production by P. aeruginosa, we applied different concentrations of inhibitor or control peptides in combination with 0.03 μg/mL ciprofloxacin. Because pyocin expression in Δ*xerC* cells is sensitive to ciprofloxacin, we reasoned that treatment with a fluoroquinolone antibiotic might sensitize cells to even mild recombinase inhibition, resulting in measurable changes to pyocin expression. As expected, treatment with the control peptide over a range of 0.1 to 50 μM impacted neither culture growth nor pyocin expression (Fig. 7C and E). However, cotreatment with the WRWYCR inhibitor at 10 or 50 μM, but not at 0.1 μM, yielded modest changes to the magnitude and timing of pyocin expression. A 10 μM concentration of inhibitor induced an earlier and higher peak of pyocin expression relative to that of ciprofloxacin alone (Fig. 7C and D) or inhibitor alone (Fig. 7A and B). Cotreatment with 50 μM inhibitor induced a later peak of pyocin expression, in accord with initial inhibition of growth (Fig. 7C). As with 10 μM inhibitor cotreatment, the peak of pyocin expression was greater than that for either treatment alone (Fig. 7C and D, compare Fig. 6A and B). These data suggest that recombinase inhibition by hexapeptides like WRWYCR can modestly impact pyocin expression in cells with an already-active SOS response. However, the slightly greater peak pyocin expression induced by addition of peptide inhibitor to ciprofloxacin remained severalfold lower than the peak observed for ciprofloxacin treatment of Δ*xerC* cells (Fig. 7D). Collectively, our results imply that peptide recombinase inhibitors do not substantially inhibit XerC in a manner leading to pyocin expression.

## DISCUSSION

We derive four principal findings from our study. First, the absence of XerC not only results in RecA-independent elevation of basal pyocin expression but also permits substantial additional stimulation of pyocin expression by ciprofloxacin or MMC (Fig. 1 and 2). This additional stimulation is likewise RecA independent despite being provoked by agents that cause DNA damage. Nonetheless, under all tested conditions, pyocin expression requires PrtN (Fig. 3). Second, heterogeneity in pyocin expression across individual cells is preserved even under very strong bulk expression (Fig. 4), consistent with a robust system to prevent widespread cell lysis. Third, specific inhibition of XerC recombinase activity induces pyocin expression only via the canonical RecA-dependent pathway (Fig. 5), implying that XerC has a second function in pyocin regulation that is separate from its recombinase activity. Finally, a previously characterized bacterial tyrosine recombinase inhibitor does not activate pyocin expression at either inhibitory or subinhibitory concentrations (Fig. 6 and 7).

Our finding that deletion of *xerC* raises basal pyocin expression levels that can be further stimulated by ciprofloxacin or MMC helps to explain the previously observed hypersensitivity of Δ*xerC* cells to ciprofloxacin (12). It also raises additional questions with respect to the nature of the RecA-independent pathway for pyocin expression. RecA-independent induction of typically RecA-dependent pathways is not entirely without precedent. Expression of certain capsular polysaccharide synthesis regulators can induce RecA-independent lambda prophage induction in E. coli (21), and mycobacteria have a well-studied RecA-independent DNA damage response that is regulated by proteasome accessory factors (22). Irrespective of the pathway, PrtN appears to be strictly required for pyocin expression (Fig. 3), implying that *prtN* expression can occur even without activated RecA-mediated cleavage of PrtR. Identifying the factors required for RecA-independent pyocin induction is an important future goal.

Our microscopic analysis showed that even under DNA damage-inducing conditions producing the strongest pyocin response, pyocin gene expression remained highly heterogeneous, with fewer than half of cells showing detectable expression (Fig. 4). Clearly, when inducing a gene cluster that encodes a lethal holin and lysin, a heterogeneous response can be advantageous, as nonexpressing cells are protected from lysis. It will be interesting to uncover the basis for the strong heterogeneity of pyocin production, which remains unclear.

We were initially surprised to find that the modest elevation of pyocin expression in strains bearing the recombinase-inactive XerC_Y272F_ variant was mediated by RecA (Fig. 5). However, distinct mechanisms are concordant with the stronger pyocin expression seen in Δ*xerC* strains relative to *xerC*_Y272F_ strains (12). These results also imply that the presence of recombinase-dead XerC in cells provokes RecA activation in a way that the complete absence of XerC does not. Moreover, the absence of RecA-independent pyocin expression in *xerC*_Y272F_ cells suggests that XerC has a second function in pyocin regulation that is not affected by its enzymatic inactivation. One intriguing possibility warranting further investigation is that XerC also acts as a transcriptional regulator at sites other than the *dif* sites at which it cooperates with XerD to achieve recombination (14).

Because deletion of *xerC* leads to increased pyocin production and sensitizes cells to ciprofloxacin, a member of the clinically important fluoroquinolone class of antibiotics, we considered it important to test whether drug treatment could achieve a similar effect. To our knowledge, the only known inhibitors of bacterial recombinases are hexapeptides, which have primarily been characterized in E. coli. These inhibitors, the best known of which is WRWYCR (or wrwycr, constructed from d-amino acids), trap Holliday junctions (including intermediates in XerCD-mediated chromosome dimer resolution), can prevent prophage excision, and thereby inhibit bacterial growth (18–20). We confirmed that WRWYCR, but not a previously described control hexapeptide, WKHYNY (19), effectively inhibited growth of our P. aeruginosa strains (Fig. 6). Neither our experiments nor previous work (19) rules out the possibility that the control WKHYNY peptide is simply not taken up by bacterial cells. Nonetheless, given that genetic inactivation of XerC (XerC_Y272F_) induced RecA-dependent pyocin expression (Fig. 5) and that DNA damage stemming from wrwycr-mediated inhibition induced the SOS response in E. coli (19), we expected that general recombinase inhibition might induce pyocins. However, we observed no induction under growth-inhibitory levels of peptide treatment (Fig. 6). Because we noticed that relatively low, sublethal concentrations of ciprofloxacin (0.03 μg/mL) resulted in more frequent pyocin-ON cells than did the higher concentrations (1 μg/mL) we used previously (12), we also tested lower concentrations of inhibitor peptide that had only weak effects on bacterial growth. We never observed pyocin induction, confirming that recombinase inhibition via Holliday junction trapping does not, on its own, induce pyocin expression. Even in combination with ciprofloxacin treatment, inhibitor peptides had only a modest impact on pyocin expression and timing (Fig. 7).

Collectively, our results highlight the existence of an alternative, RecA-independent but DNA damage-inducible pathway for pyocin expression that we observe only in *xerC*-deleted strains. Further, these findings imply that P. aeruginosa is capable of sensing DNA damage even without RecA. Many questions remain. What regulatory elements and proteins comprise the alternative pathway, how does it sense DNA damage, and what other genes besides those encoding the R/F pyocins are under its control? What is the role of XerC in regulating the alternative pathway? Is pyocin induction by other stressors, such as oxidative stress (7), affected by the absence of XerC? Can pharmaceutical inhibition of XerC be achieved to activate the alternative pathway and sensitize P. aeruginosa to fluoroquinolone antibiotics? We look forward to tackling these mysteries.

## MATERIALS AND METHODS

### Strains and growth conditions.

Escherichia coli SM10 and Pseudomonas aeruginosa PA14 were grown in Luria-Bertani (LB) Lennox broth (10 g/L tryptone, 5 g/L yeast extract, 5 g/L NaCl) or on LB agar plates fortified with 1.5% Bacto agar at 37°C. When appropriate, 25 μg/mL irgasan (to specifically select for P. aeruginosa) plus 75 μg/mL tetracycline, 25 μg/mL irgasan plus 75 μg/mL gentamicin, 25 μg/mL tetracycline, or 20 μg/mL gentamicin was added to liquid or solid media. P. aeruginosa was also selected over E. coli for some strains by growth on VBMM containing citrate as the sole carbon source (23). The strains used in this work are listed in Table 1 and in Table S1 in the supplemental material. Markerless deletions were generated using the pEXG2 vector with counterselection on no-salt LB plates containing 15% sucrose (23) and were screened by colony PCR for the presence of deletions. Reporter strains were constructed by integration of the mini-CTX-1-gfp vector at the neutral chromosomal *attB* locus. Modes of strain and plasmid construction are given in the supplemental material. Strains deleted for *recA* were additionally phenotypically screened for their inability to enact generalized recombination by failure to generate gentamicin-resistant EXG2 transconjugants.

**TABLE 1:** Strains used in this work

Strain	Genotype or description	Reference or source
MTC2252	PA14 Δ*xerC attB*::*CTX-1-*P*_07990_*-*gfp*	12
MTC2277	PA14 *attB*::*CTX-1-*P*_07990_*-*gfp*	12
MTC2280	PA14 *attB*::*CTX-1-*P*_07990_*-*lux*	12
MTC2291	PA14 Δ*xerC* Δ*recA attB*::*CTX-1-*P*_07990_*-*gfp*	12
MTC2297	PA14 Δ*xerC attB*::*CTX-1-*P*_07990_*-*lux*	12
MTC2298	PA14 Δ*xerC* Δ*prtN attB*::*CTX-1-*P*_07990_*-*gfp*	12
MTC2301	PA14 Δ*xerC* Δ*recA attB*::*CTX-1-*P*_07990_*-*lux*	12
MTC2302	PA14 Δ*recA attB*::*CTX-1-*P*_07990_*-*lux*	12
MTC2303	PA14 Δ*prtN attB*::*CTX-1-*P*_07990_*-*lux*	This study
MTC2339	PA14 *xerC*_Y272F_ *attB*::*CTX-1-*P*_07990_*-*lux*	12
MTC2441	PA14 *xerC*_Y272F_ Δ*recA*	This study
MTC2444	PA14 *xerC*_Y272F_ Δ*recA attB*::*CTX-1-*P*_07990_*-*lux*	This study
MTC2448	PA14 Δ*recA attB*::*CTX-1-*P*_07990_*-*gfp*	This study

### Growth curve and kinetic luciferase assays.

Strains of interest were grown on LB plates overnight, and single colonies were inoculated into LB liquid broth with appropriate antibiotics and grown overnight with shaking at 37°C. Strains were then diluted 1,000-fold into fresh LB medium and grown to early exponential phase (2 to 4 h). The cultures were then mixed in 1.5-mL microcentrifuge tubes with stocks of ciprofloxacin, mitomycin C (both in sterile water), or hexapeptides (in dimethyl sulfoxide [DMSO]) at >50× the final concentration and aliquoted (200 μL) into wells of a clear-bottomed, opaque white 96-well plate to generate technical replicates (3 to 4 per biological replicate). The plate was incubated in a BioTek Synergy H1 plate reader (BioTek, USA) at 37°C for 20 h with double-orbital shaking. OD at 600 nm (OD_600_) and luminescence (gain = 135, integration time, 1 s) measurements were obtained every 10 min. At least 3 biological replicates were assayed for each combination of strain and condition. Results were analyzed in MS Excel and plotted using GraphPad Prism.

### Fluorescence microscopy.

Strains of interest were grown in 3 mL of LB liquid broth with appropriate antibiotics overnight. The cultures were then diluted 1,000-fold in fresh LB and grown to early exponential phase (3 to 4 h). The cultures were split, and 0.03 μg/mL ciprofloxacin was added (or not) to cells and incubated for a further 135 min before imaging. Cells were immobilized by spotting 0.5 μL of the growing culture onto an LB-agarose pad and covering with cover glass. Imaging was immediately performed using a Nikon Eclipse Ti inverted fluorescence microscope with a Photometrics Prime 95B scientific complementary metal oxide semiconductor (sCMOS) digital camera, a Lumencor Sola SE II 365 LED Light Engine, and an OKO temperature-controlled enclosure. Cell images were captured at ×100 magnification in both phase and GFP channels. For quantification of GFP-positive cells, images were analyzed as in our prior work (12) using the MicrobeJ plugin for ImageJ (24), segmenting on phase contrast and taking the mean GFP values of the corresponding fluorescence images. Segmentation was performed with default values except that minimum and maximum areas of 100 and 400 px were used, and circularity was delimited from 0 to 0.9. For options, “exclude on edges,” “shape descriptors,” “segmentation,” and “intensity” were selected. A threshold of 1.2 times the average background fluorescence was selected to denote GFP positivity, as 100% of PA14 cells without a GFP reporter fell below this threshold, which was approximately 5.5 standard deviations above the mean fluorescence of reporter-free cells (12). Analyses were conducted using MS Excel and plotted using GraphPad Prism.

## References

[1] Ge P, Scholl D, Prokhorov NS, Avaylon J, Shneider MM, Browning C, Buth SA, Plattner M, Chakraborty U, Ding K, Leiman PG, Miller JF, Zhou ZH. 2020. Action of a minimal contractile bactericidal nanomachine. Nature 580:658–662. doi:10.1038/s41586-020-2186-z. 32350467 PMC7513463

[2] Michel-Briand Y, Baysse C. 2002. The pyocins of *Pseudomonas aeruginosa*. Biochimie 84:499–510. doi:10.1016/S0300-9084(02)01422-0. 12423794

[3] Oluyombo O, Penfold CN, Diggle SP. 2019. Competition in biofilms between cystic fibrosis isolates of *Pseudomonas aeruginosa* is shaped by R-pyocins. mBio 10:e01828-18. doi:10.1128/mBio.01828-18. 30696740 PMC6355985

[4] Scholl D. 2017. Phage tail-like bacteriocins. Annu Rev Virol 4:453–467. doi:10.1146/annurev-virology-101416-041632. 28961412

[5] Waite RD, Curtis MA. 2009. *Pseudomonas aeruginosa* PAO1 pyocin production affects population dynamics within mixed-culture biofilms. J Bacteriol 191:1349–1354. doi:10.1128/JB.01458-08. 19060137 PMC2631993

[6] Nakayama K, Takashima K, Ishihara H, Shinomiya T, Kageyama M, Kanaya S, Ohnishi M, Murata T, Mori H, Hayashi T. 2000. The R-type pyocin of *Pseudomonas aeruginosa* is related to P2 phage, and the F-type is related to lambda phage. Mol Microbiol 38:213–231. doi:10.1046/j.1365-2958.2000.02135.x. 11069649

[7] Chang W, Small DA, Toghrol F, Bentley WE. 2005. Microarray analysis of *Pseudomonas aeruginosa* reveals induction of pyocin genes in response to hydrogen peroxide. BMC Genomics 6:115. doi:10.1186/1471-2164-6-115. 16150148 PMC1250226

[8] Cirz RT, O’Neill BM, Hammond JA, Head SR, Romesberg FE. 2006. Defining the *Pseudomonas aeruginosa* SOS response and its role in the global response to the antibiotic ciprofloxacin. J Bacteriol 188:7101–7110. doi:10.1128/JB.00807-06. 17015649 PMC1636241

[9] Matsui H, Sano Y, Ishihara H, Shinomiya T. 1993. Regulation of pyocin genes in *Pseudomonas aeruginosa* by positive (*prtN*) and negative (*prtR*) regulatory genes. J Bacteriol 175:1257–1263. doi:10.1128/jb.175.5.1257-1263.1993. 8444788 PMC193209

[10] Morse SA, Vaughan P, Johnson D, Iglewski BH. 1976. Inhibition of *Neisseria gonorrhoeae* by a bacteriocin from *Pseudomonas aeruginosa*. Antimicrob Agents Chemother 10:354–362. doi:10.1128/AAC.10.2.354. 825024 PMC429747

[11] Brazas MD, Hancock RE. 2005. Ciprofloxacin induction of a susceptibility determinant in *Pseudomonas aeruginosa*. Antimicrob Agents Chemother 49:3222–3227. doi:10.1128/AAC.49.8.3222-3227.2005. 16048929 PMC1196232

[12] Baggett NS, Bronson AS, Cabeen MT. 2021. SOS-independent pyocin production in *P. aeruginosa* is induced by XerC recombinase deficiency. mBio 12:e02893-21. doi:10.1128/mBio.02893-21. 34809462 PMC8609362

[13] Blakely G, Colloms S, May G, Burke M, Sherratt D. 1991. Escherichia coli XerC recombinase is required for chromosomal segregation at cell division. New Biol 3:789–798. 1931824

[14] Blakely G, May G, McCulloch R, Arciszewska LK, Burke M, Lovett ST, Sherratt DJ. 1993. Two related recombinases are required for site-specific recombination at dif and cer in E. coli K12. Cell 75:351–361. doi:10.1016/0092-8674(93)80076-Q. 8402918

[15] Blakely GW, Davidson AO, Sherratt DJ. 2000. Sequential strand exchange by XerC and XerD during site-specific recombination at dif. J Biol Chem 275:9930–9936. doi:10.1074/jbc.275.14.9930. 10744667

[16] Hofte M, Dong Q, Kourambas S, Krishnapillai V, Sherratt D, Mergeay M. 1994. The sss gene product, which affects pyoverdin production in *Pseudomonas aeruginosa* 7NSK2, is a site-specific recombinase. Mol Microbiol 14:1011–1020. doi:10.1111/j.1365-2958.1994.tb01335.x. 7715441

[17] Grindley ND, Whiteson KL, Rice PA. 2006. Mechanisms of site-specific recombination. Annu Rev Biochem 75:567–605. doi:10.1146/annurev.biochem.73.011303.073908. 16756503

[18] Gunderson CW, Boldt JL, Authement RN, Segall AM. 2009. Peptide WRWYCR inhibits the excision of several prophages and traps Holliday junctions inside bacteria. J Bacteriol 191:2169–2176. doi:10.1128/JB.01559-08. 19181810 PMC2655501

[19] Gunderson CW, Segall AM. 2006. DNA repair, a novel antibacterial target: Holliday junction-trapping peptides induce DNA damage and chromosome segregation defects. Mol Microbiol 59:1129–1148. doi:10.1111/j.1365-2958.2005.05009.x. 16430689

[20] Kepple KV, Boldt JL, Segall AM. 2005. Holliday junction-binding peptides inhibit distinct junction-processing enzymes. Proc Natl Acad Sci USA 102:6867–6872. doi:10.1073/pnas.0409496102. 15867153 PMC1100769

[21] Rozanov DV, D’Ari R, Sineoky SP. 1998. RecA-independent pathways of lambdoid prophage induction in Escherichia coli. J Bacteriol 180:6306–6315. doi:10.1128/JB.180.23.6306-6315.1998. 9829941 PMC107717

[22] Muller AU, Imkamp F, Weber-Ban E. 2018. The mycobacterial LexA/RecA-independent DNA damage response is controlled by PafBC and the Pup-proteasome system. Cell Rep 23:3551–3564. doi:10.1016/j.celrep.2018.05.073. 29924998

[23] Hmelo LR, Borlee BR, Almblad H, Love ME, Randall TE, Tseng BS, Lin C, Irie Y, Storek KM, Yang JJ, Siehnel RJ, Howell PL, Singh PK, Tolker-Nielsen T, Parsek MR, Schweizer HP, Harrison JJ. 2015. Precision-engineering the *Pseudomonas aeruginosa* genome with two-step allelic exchange. Nat Protoc 10:1820–1841. doi:10.1038/nprot.2015.115. 26492139 PMC4862005

[24] Ducret A, Quardokus EM, Brun YV. 2016. MicrobeJ, a tool for high throughput bacterial cell detection and quantitative analysis. Nat Microbiol 1:16077. doi:10.1038/nmicrobiol.2016.77. 27572972 PMC5010025

