# Supplementary material for: DNA Damage-Inducible Pyocin Expression Is Independent of RecA in xerC-Deleted Pseudomonas aeruginosa
Source: Microbiol Spectr. 2022 Jun 16;10(4):e01167-22. doi: 10.1128/spectrum.01167-22 (PMC9431673; doi:10.1128/spectrum.01167-22)
Supplement: Supplemental file 1 — Supplemental material. Download spectrum.01167-22-s0001.pdf, PDF file, 0.08 MB [file spectrum.01167-22-s0001.pdf]

Table S1. ***Escherichia coli*** **strains used in this work.**

Strain	Genotype or description	Source or reference
MTC2179	SM10 pEXG2-Δ*recA*, Gent^R	(1)
MTC2286	SM10 pCTX-1-P$_{07990}$-gfp, Tet^R	(1)
MTC2287	SM10 pCTX-1-P$_{07990}$-lux, Tet^R	(1)

Modes of strain construction

P. aeruginosa strains

MTC2441

MTC2327 (PA14 *xerC*$_{Y272F}$) (1) was mated with MTC2179: 1.2 ml of an overnight LB culture of MTC2179 was mixed with 350 µl of an overnight LB culture of MTC2327, centrifuged for 2 min at 10,000 *g*, the supernatant was discarded, and the concentrated cell suspension was spotted on an LB-1.5% agar plate and dried. The plate was incubated at 30°C overnight, and the resulting colony was scraped up with a sterile loop and resuspended in 500 µL sterile phosphate-buffered saline (PBS) or LB. An aliquot (typically 100 µL) of the suspension was spread on VBMM agar containing 75 µg/ml gentamycin and grown overnight at 37°C to select for *P. aeruginosa* transconjugants with integrated pEXG2-Δ*recA*, and several of the resulting colonies were streaked onto no-salt LB with 15% sucrose to select second crossovers (2). A number of the sucrose-resistant colonies arising were then patched on LB and LB with 20 µg/mL gentamycin. At least 2 sucrose-resistant, gent-sensitive clones were then streaked for single colonies, checked by PCR for presence of the desired deletion, and frozen at -80°C in 25% glycerol.

MTC2444

MTC2441 was mated with MTC2287 as described above for MTC2441, and a 10-µL aliquot of the resuspended mating colony was spread on LB plates with 75 µg/mL tetracycline and 25 µg/mL irgasan to select for *P. aeruginosa* transformants. At least 2 colonies were then re-streaked for single colonies on LB-tet (25 µg/mL), grown in LB overnight at 37°C, and stored in 25% glycerol at -80°C.

MTC2448

Made as for MTC2444, except MTC 2274 (PA14 Δ*recA*) (1) was mated with MTC2286.

MTC2303

Made as for MTC2444, except MTC 2276 (PA14 Δ*prtN*) (1) was mated with MTC2287.

References

1. Baggett NS, Bronson AS, Cabeen MT. 2021. SOS-Independent Pyocin Production in P. aeruginosa Is Induced by XerC Recombinase Deficiency. mBio 12:e0289321.
2. Hmelo LR, Borlee BR, Almblad H, Love ME, Randall TE, Tseng BS, Lin C, Irie Y, Storek KM, Yang JJ, Siehnel RJ, Howell PL, Singh PK, Tolker-Nielsen T, Parsek MR, Schweizer HP, Harrison JJ. 2015. Precision-engineering the Pseudomonas aeruginosa genome with two-step allelic exchange. Nat Protoc 10:1820-41.
